# Young people’s perceptions of the challenges and opportunities during the Mainland China–Hong Kong convergence

**DOI:** 10.1057/s41599-023-01755-2

**Published:** 2023-06-07

**Authors:** T. Wing Lo, Gloria Hongyee Chan, Gabriel Kwun Wa Lee, Xin Guan, Sharon Ingrid Kwok

**Affiliations:** 1 Caritas Institute of Higher Education, Hong Kong, China; 2 City University of Hong Kong, Hong Kong, China; 3 Western Sydney University, Sydney, Australia

**Keywords:** Sociology, Cultural and media studies, Social policy

## Abstract

Since the handover of the sovereignty of Hong Kong from Britain to China in 1997, convergence between Mainland China and Hong Kong has gradually emerged. During this process, young people have engaged in demonstrations to express their dissatisfaction with government policies and limited socio-economic progression. However, the underlying reasons for their dissatisfaction have not been fully investigated. This study investigates their perceived challenges and opportunities during the convergence, with the objective of identifying the factors affecting the Mainland China–Hong Kong convergence and examining young people’s perceived challenges and opportunities during the convergence. Mixed research methods of focus groups and a survey were adopted. Ten focus groups with 83 participants were conducted to collect qualitative data on the factors relating to convergence. Based on the qualitative data, a questionnaire was constructed to investigate young people’s perceived challenges and opportunities during the convergence, using a sample of 1253 young people. Ordinary least-squares regression was applied to analyse the relationships among identified factors. The study found that Hong Kong’s youth tended to regard the Mainland China–Hong Kong convergence as an opportunity for socio-economic progression, and they identified three challenges during the convergence. It also revealed that young people’s higher education, perceived housing challenges, and perceived socio-economic challenges are negatively related to the convergence, whereas their perceived challenges associated with entrepreneurship and innovation are positively related to the convergence. The development of more well-balanced and mutually beneficial policies that satisfy the needs of young people will lead to a higher acceptance of the convergence. As such, young people will be more willing to embrace the opportunities and face the challenges brought about by the convergence, resulting in a more harmonious society and socio-economic progression.

## Introduction

Britain handed over the sovereignty of Hong Kong to China in 1997. Under the principle of “One Country Two Systems”, convergence between Mainland China and Hong Kong has gradually emerged, and this has brought about a challenging social environment in Hong Kong (Day and Fiske, 2017). During this process, growing controversies in social and political issues have emerged, resulting in several mass demonstrations against government policies since 2011 (Lee and Lo, 2020). The government believes that, in addition to political factors, the causes of these demonstrations are closely related to internal and external social, economic, and environmental factors, such as the Mainland China-Hong Kong convergence (CHC), Hong Kong’s economic transformation, and global economic instability (Information Services Department, 2019). In the face of fierce competition inside and outside Hong Kong, young people have felt powerless and hesitant about the future (Day and Fiske, 2017), and have engaged in demonstrations to express their dissatisfaction with social inequalities and limited socio-economic progression (Kearney and Levine, 2016). There is, however, a lack of interpretation of the views of young people in Hong Kong on the factors facilitating and inhibiting the CHC. Against this backdrop, this study attempts to investigate young people’s perceived opportunities and challenges from 1997 to 2017 during the CHC so as to uncover the roots of their social dilemma and the conflicts underlying such a convergence. The findings will provide insights into the development of public policies that facilitate convergence.

## Literature review

### Education

Education has always been treated as a key to personal success (Machin et al., 2012). However, because of a shortage of local government-funded university places, fewer than 23% of Hong Kong students can be enrolled in an undergraduate degree (Mok and Neubauer, 2016). Among the admitted undergraduates, students from low-income families make up <15% of the enrolment rate, which is 3.7 times lower than the rate for students from rich families (O’Sullivan and Tsang, 2015). As a result, the current education system implicitly marginalises and excludes students from lower social classes, resulting in a vicious cycle of social and intergenerational mobility (O’Sullivan and Tsang, 2015).

Traditionally, the level of education has been tied to professional jobs, but this relationship has now been broken. Chiu and Yan (2015) have pointed out that tertiary education graduates only receive an income similar to secondary school graduates. In other words, higher education has not led to upward socio-economic mobility in Hong Kong. Alongside the substantial increase in associate degree places after the handover, companies have gradually raised their thresholds for recruitment (Wong and Koo, 2016), and thus young people face fiercer competition in career paths.

Furthermore, service sectors have continued to expand after the handover, resulting in monotonous development, and only a few professionals can achieve high salaries (Chiu and Yan, 2015; Wong and Koo, 2016). Since the handover, the monotonous development of industrial and economic structures has brought fewer opportunities for young people to work in middle-class jobs and to obtain educational advantages in their careers (Chiu and Yan, 2015). The vacancies in middle-class jobs have been at a standstill, but the number of lower-class jobs has continued to grow, resulting in a block in upward socio-economic mobility for young people (Chiu and Yan, 2015). Furthermore, most middle-class jobs have become less well-paid contract-basis positions rather than secure, well-paid tenure ones (Wong and Koo, 2016). A growing number of Hong Kong citizens consider themselves in the lower social class and are more pessimistic about the future (Tong and Luk, 2013). The situation of the younger generation is worse because the increase in their median income (+0.3% for ages 15–24; +1.2% for ages 25–34) is lower than that of the overall working population (+1.5% for all employees) (Census and Statistics Department; 2021; Chiu and Yan, 2015).

Even worse, graduates have to deal with hefty college loan repayment after graduation. The Hong Kong government does not offer full funding to tertiary students, so they have to bear heavy debts once they have graduated, let alone those who have followed self-financed programmes (Mok, 2016; Mok and Neubauer, 2016). The disadvantages are worse for those who borrow to pay tuition fees or living costs (Mok and Neubauer, 2016).

Consequently, university-educated young people are not in as many advantageous positions as those who are less well-educated regarding their earning abilities. Education is no longer a predictor of job opportunity, position, salary, or social status (Mok and Neubauer, 2016). As a result, the younger generation is pessimistic, frustrated, and even hopeless regarding their future.

### Housing

Since the handover, housing has become a significant concern among Hong Kong youth (Mok and Neubauer, 2016), and it continues to worsen (Shek, 2020). The reason lies in the housing policies, which affect the affordability of houses. Although Hong Kong’s private property prices declined sharply because of the financial crisis in 1997 and SARS in 2003, they demonstrated a rapid and consistent increase after these periods (Li, 2015, 2016). On top of that, the housing problem became more alarming when the government allowed non-local house buyers to gain permanent citizenship through property investment in 2015 (Li, 2016), partly contributing to the runaway private property prices (Leung et al., 2020).

On the other hand, youth income is another barrier to homeownership. The ratio of house prices to wages grew rapidly after 2004 (Leung et al., 2020). Regardless of private or public housing, youths generally have to spend over 40% of their income on housing (O’Sullivan and Tsang, 2015). The discrepancy between income and property prices makes homeownership impossible. Consequently, only 11% of young people aged 30–35 have their own property, and nearly 40% of them have to live with their parents until an unknown age (Li, 2015). Hence, many young people cannot establish their own families, which causes frustration and triggers a sense of hopelessness (Shek, 2020).

For public housing, the government has further turned its policy from providing new houses to urban renewal (Li, 2016). Meanwhile, the government’s constant insufficiency and unfulfilled supply targets put further pressure on the need for public housing (Leung et al., 2020). The government has set a stringent upper boundary on applicants’ monthly income (HK$12,800 for an individual applicant in April 2020) and total assets, which was lower than the median monthly wage of Hong Kong employees (HK$18,400; US$1 = HK$7.8) (Census and Statistics Department, 2021), implying that most ordinary employees are not eligible to apply for public housing. Hence, youths have to face the dilemma of either developing their career for a higher salary or seeking a low-paying job so as to qualify for public housing. Under these circumstances, homeownership in Hong Kong is an extravagant hope. With the challenges of purchasing a private flat or the almost-impossible eligibility in terms of applying for public housing, Hong Kong youths foresee a grim future.

During the industrialisation and rapid economic growth of the 1960s and 1970s, if individuals were sufficiently capable and industrious, they could quickly have the opportunity to move upwards (Chiu and Yan, 2015; Wong and Koo, 2016). During that period, Hong Kong people believed that individual efforts led to personal achievement and raised or improved one’s social class (Wong and Koo, 2016). Unfortunately, to date, young people have suffered unemployment, disproportionate rewards, and a heavy financial burden with respect to housing and education (Mok and Neubauer, 2016). Chiu and Yan (2015), and thus a significant fall in the number of young people with middle-class careers has been found.

### Mainland China–Hong Kong convergence (CHC)

In the past decade, several social and economic policies—including the Admission Scheme for Mainland Talents and Professionals, Quality Migrant Admission Scheme, and Immigration Arrangement for Non-local Graduates—have been implemented to speed up the CHC. An increasing number of Mainland Chinese companies have been expanding their businesses in Hong Kong, resulting in the constituent stocks of the Hong Kong Stock Exchange being mainly Chinese companies (Cabestan and Florence, 2018). Economic growth and development should allow people to attain a certain level of economic success, increase upward mobility, and strengthen social stability (Day and Fiske, 2017). However, this is not the case for Hong Kong’s youth.

Hong Kong-based Chinese companies prefer to recruit mainlanders because of their fluent Putonghua and their mainland connections, which benefit companies’ business development in the mainland market (Cabestan and Florence, 2018). An increasing number of mainland students study and reside in Hong Kong because of the higher financial rewards (Cabestan and Florence, 2018). Given the lower salaries on the mainland, more mainland university graduates seek jobs in Hong Kong, which intensifies the competition (Fung and Chan, 2017; Mok, 2016).

Moreover, the fierce competition for social resources decreases education resources (Lai Wong et al., 2015). Admission to Hong Kong universities attracts mainland talent to study and work in Hong Kong (Lai Wong et al., 2015). The statistics published by the University Grants Committee of Hong Kong (2015) show that university students from Mainland China accounted for 78% of enrolled non-local students in 2013–14. Local young people perceive this as them being deprived of educational opportunities, creating competition for scarce educational resources between mainland students and Hong Kong youth (Yu and Zhang, 2016). Moreover, some social resources have been reallocated to cater to new mainland immigrants (Lowe and Tsang, 2017; Wong and Koo, 2016). As a result, verbally hostile taunts, names, and stereotypes, such as *Ah Chaan* (uneducated or hayseed) and locust (exploiting social resources), have been applied to mainlanders (Lowe and Tsang, 2017).

The implementation of the Individual Visit Scheme, which attracts a considerable number of mainland visitors to Hong Kong, has generated disturbances, such as traffic congestion, crowding, a declining level of cleanliness in public places, and a shortage of daily goods in the market, in Hong Kong people’s lives (Chan, 2014; Fung and Chan, 2017). Under the convergence, Hong Kong has experienced drastic changes in public policies, economics, society, and culture. Some authors (Hui and Lo, 2015; Li and Lo, 2018) have even used the term “mainlandization” to describe the process of Mainland China absorbing Hong Kong. The people of Hong Kong have experienced an invasion shock—have felt astonished, offended, and overwhelmed—by the influx of mainland visitors, who have retained their culture and moral codes (Pyvis and Chapman, 2005), and thus regarded mainlanders as threatening invaders. This has resulted in the development of localism and hatred towards the invasion by mainlanders, which have arisen from their worry about their middle-class dream being in jeopardy (Lowe and Tsang, 2017). The identity of “Hongkongers” has been crystallised through a psychological process of disidentification with China because of the differences in language, culture, social behaviours, politics, and historical experiences (Central Policy Unit, 2015; Lowe and Tsang, 2017).

## Methodology

The above literature demonstrates that Hong Kong’s youth have faced a lot of challenges after the convergence. Along with increasing social demonstrations in Hong Kong, the government and some observers believe that the active political engagement of Hong Kong’s youth is a result of blocked socio-economic progression, whereas some think that the CHC could be an opportunity for them (Chiu and Yan, 2015; Mok and Neubauer, 2016; Sheng, 2013). Against this backdrop, the main objective of this study is to identify the factors that affect the CHC and examine young people’s perceived opportunities and challenges during the convergence.

Given the CHC, the principle of “One Country Two Systems” is unique to China, and as the social and political situations between the mainland and Hong Kong are complex, we cannot identify existing tools with validated items to measure such uniqueness. Thus, the present study adopted a mixed-method design. First, the qualitative method using focus groups with a small sample was used to identify grounded items to reveal young people’s perspectives on the research topic. Second, based on the qualitative data, a questionnaire was constructed to survey a large sample of young people and confirm their perspectives on the opportunities and challenges during the CHC. Informed consent from each participant was obtained in both phases.

### Focus groups

The focus groups aimed to understand young people’s subjective perceptions toward the social, economic, and political situations related to the CHC. Ten focus groups were conducted from March to May 2017 (see Table 1). Each group lasted approximately two hours. Altogether, 83 participants attended the focus groups, with an average of 8.3 participants per group. Through purposive sampling, only permanent Hong Kong residents were recruited and the participants were selected from different age groups, occupations, social and political backgrounds, and genders (see Table 2). Each participant received a HK$200 [US$25] supermarket coupon as an incentive.

**Table 1:** Summary of the focus group participants.

	Number of participants	Participant code
Focus Group 1	8	1.1–1.8
Focus Group 2	10	2.1–2.10
Focus Group 3	9	3.1–3.9
Focus Group 4	10	4.1–4.10
Focus Group 5	9	5.1–5.9
Focus Group 6	8	6.1–6.8
Focus Group 7	5	7.1–7.5
Focus Group 8	6	8.1–8.6
Focus Group 9	9	9.1–9.9
Focus Group 10	9	10.1–10.9

**Table 2:** Demographics of focus group participants.

Demographics	Number of participants
*Gender*	
Male	41
Female	42
*Age*	
18–19	5
20–24	26
25–29	24
30–34	19
35–39	9
*Backgrounds*	
Self-employed/freelance	8
Entrepreneur	8
Professional (e.g. doctor, lawyer, accountant, architect/surveyor, social worker, teacher)	8
Frontline staff (e.g. paraprofessional/clerical support staff/frontline service worker and shop sales staff)	8
Middle management (e.g. manager, executive)	8
Member of political party or activist	8
Government-funded undergraduate or postgraduate student	8
Self-financing undergraduate, postgraduate or sub-degree student	8
Not in education, employment, or training (NEET) youth	11
Elite youth (Hong Kong Outstanding Teens Election awardees)	8
Total	83

To collect diversified views, participants represented different political views, occupations, schools, and courses. For example, the respondents comprising “political activists and party activists” included political parties and social activists from different political spectrums, including pro-government parties, pro-democracy camps, and young people who had participated in social movements or run for elections. As for students, participants were recruited from different educational institutions and academic programs. Professionals were also recruited from different fields, including medicine, surveying, accounting, education, and social workers. Participants were chosen based on these background categories and mixed in each focus group, with the purpose of gathering views from different perspectives and facilitating interaction between participants from diversified backgrounds. As shown in Table 2, half of the participants were male, and the other half were female. The majority were between the ages of 20 and 34 (83%). The number of participants was equally divided (8 participants each) among self-employed or freelance workers, entrepreneurs, professionals, frontline workers, middle-management staff, members of political parties, college students, and elite youth, while 11 participants were not in education, employment, or training (NEET).

The focus group discussions consisted of open-ended questions and predefined questions, that covered the CHC, housing, education, the economy and business, employment, culture, politics and society, and social cleavages, allowing participants to freely put forward their views on these issues. The discussions were audio-recorded with the participant’s consent. All the recordings were transcribed into text documents by a research assistant and confirmed by the group leader. Textual analysis of the transcripts with line-by-line coding was adopted to generalise the themes. Responses with high similarity were organised under the same code. Thereafter, consistent reflexivity was conducted throughout the analysis so that each theme was consistently reflected and reviewed with the transcripts. Eventually, four main themes were summarised (see Table 3), which represent the challenges and opportunities during the CHC as perceived by the youth participants.

**Table 3:** Themes and subthemes identified in the focus groups.

	Main themes	Subthemes
Challenges	1. Challenges associated with entrepreneurship and innovation	
	2. Housing challenges	
	3. Socio-economic challenges	I. Social challenges
		II. Economic challenges
Opportunities	1. Mainland China–Hong Kong Convergence	I. Acceptance of Chinese identity
		II. Acceptance of cross-border living and working
		III. Acceptance of convergence

### Survey

The results from the focus groups were analysed and synthesised to construct a survey questionnaire, which could be completed in 15–20 min. A pilot study was conducted to examine the reliability and feasibility of the draft questionnaire. Each participant received a HK$50 supermarket coupon [US$6] as an incentive to complete the self-administered questionnaire. The target participants were young people of different ages and social strata. To reach out to them more widely, the survey was conducted in three ways: (1) on various university campuses, (2) in food courts and other high-traffic areas, and (3) by email and online questionnaires. On-campus surveys were conducted during the daytime and evenings to reach all students. Regarding the food courts, 19 locations across Hong Kong Island, Kowloon, and the New Territories were surveyed. Furthermore, to cater to the needs of NEET youth, email and online surveys were used to reach them. A total of 1253 valid responses were collected from July to September 2017.

The participants’ socio-demographic details are listed in Table 4. Among the participants, 552 (44.1%) were male, and 701 (55.9%) were female, with a mean age of 27.2 years (standard deviation = 6.8 years); more than a quarter (26.4%) were married, about half (48.7%) had attained a bachelor degree education or higher, and about one-third (32.4%) lived in rented housing. The classification of occupations followed the International Standard Classification of Occupations (ISCO) adopted by the Census and Statistics Department (2016).

**Table 4:** Demographics of survey participants (*N* = 1253).

Demographics	Number of participants	Percentage
*Gender*		
Male	552	44.1
Female	701	55.9
*Age*		
18–19	249	19.9
20–24	248	19.8
25–29	261	20.8
30–34	253	20.2
35–40	242	19.3
*Marital status*		
Married	331	26.4
Unmarried	922	73.6
*Monthly income*		
Below HK$10,000 (US$1 = HK$7.8)	436	35.6
HK$10,000–19,999	341	27.8
HK$20,000–29,999	228	18.6
HK$30,000–39,999	105	8.6
HK$40,000–49,999	40	3.6
HK$50,000–59,999	27	2.2
HK$60,000–69,999	15	1.2
HK$70,000–79,999	13	1.1
HK$80,000 or above	21	1.7
*Education attainment*		
Grade 6 or below	4	0.3
Grade 7–9	26	2.1
Grade 10–11	178	14.2
Grade 12–13	229	18.3
Higher Diploma or Associate Degree	206	16.5
Bachelor’s degree	473	37.8
Master’s degree or above	136	10.9
*Type of housing*		
Owned Housing	843	67.6
Rented Housing	405	32.4
*Occupations*		
Group I Occupation	440	35.1
Group II Occupation	441	35.2
Full-time tertiary student	326	26.0
Unemployed	46	3.7

**Fig. 1:**
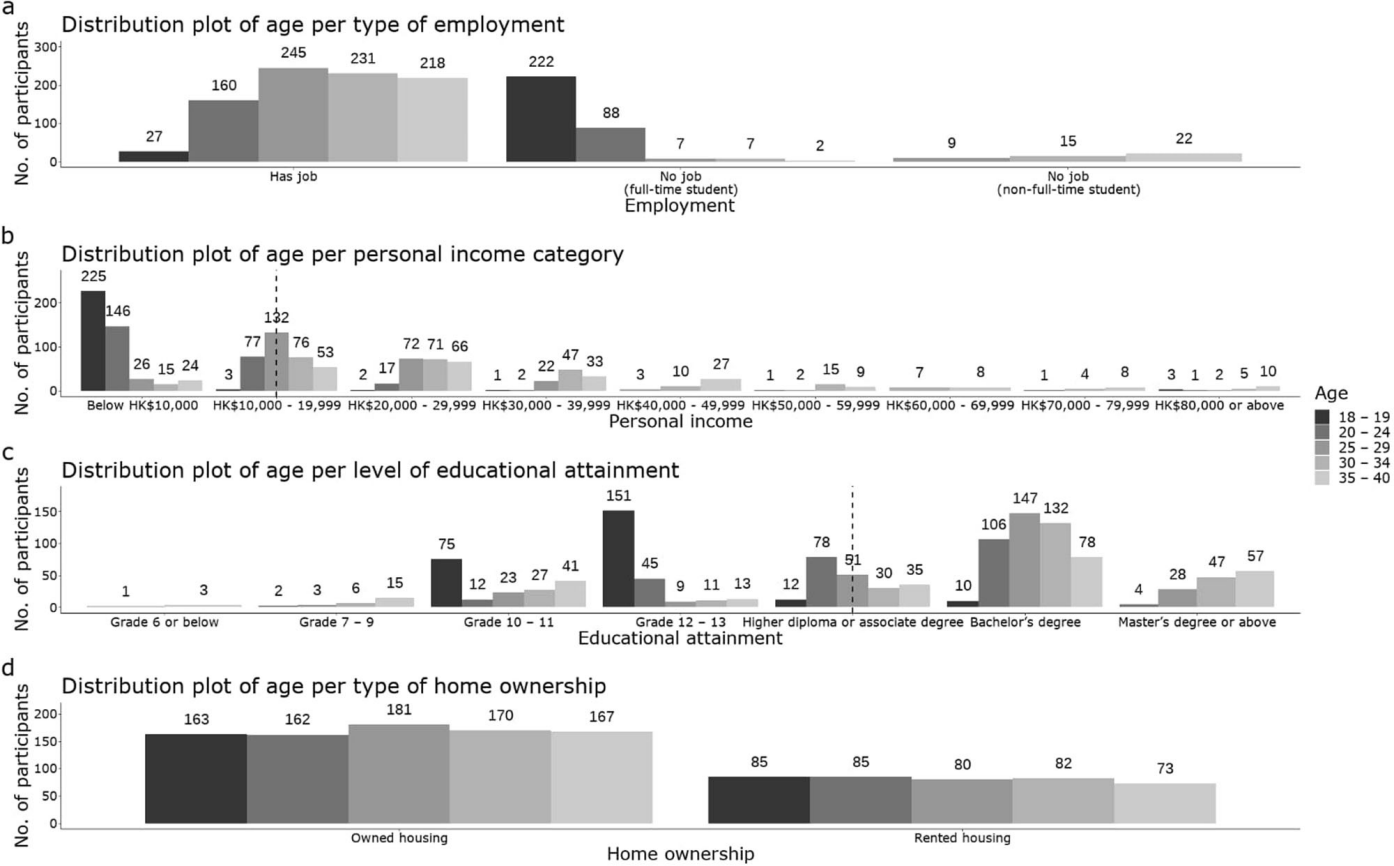
Distribution of age per level of socio-demographic feature. **a**–**d** The age distribution by employment, personal income, educational attainment, and homeownership, respectively. The darker the color of the bars, the younger the age of the group represented. Note: The black dashed line in (**b**) and (**c**) refers to the median value of personal income and educational attainment.

Figure 1 presents the distribution of participants’ age by levels of four other socio-demographic features. Except for home ownership, which has an equivalent age distribution in the owned housing group and the rented housing group (Fig. 1d), Fig. 1a–c share different age distributions between and within groups in response to employment, personal income, and education attainment. For employment status (Fig. 1a), most participants had a job by the time they received the survey, and young and full-time students accounted for a large proportion of those who currently have no job status. For personal income (Fig. 1b), younger participants’ (age 18–19) income is concentrated in the lowest bracket, and they comprise the main population of this bracket. Moving up to the next one or several income brackets, young people aged from 20 to 40 become the main population of these brackets. Although a wide range of incomes attained by participants exists, the median income level is HK$10,000 to 19,999, which is an income level that can be considered a general income profile for the overall sample; an income lower than this can be considered a low income. For educational attainment (Fig. 1c), the distribution of age groups reveals that the majority of younger participants (age 18–19) had the highest education level of Grades 10–13, which is consistent with the education programmes in Hong Kong from primary to tertiary level.

### Hypotheses

Based on the above literature review and the four themes identified from the focus groups, the following four hypotheses were set:

**Hypothesis 1 (H1):** Perceptions of housing challenges (HC) are negatively related to the CHC. That is, the more the young people perceived challenges in their housing conditions, the lower their acceptance of the CHC would be.

**Hypothesis 2 (H2):** Higher education is negatively related to the CHC. That is, the higher the young people’s level of education, the lower their acceptance of the CHC would be.

**Hypothesis 3 (H3):** Perceptions of socio-economic challenges (SEC) are negatively related to the CHC. That is, the more the young people perceived challenges in their socio-economic conditions, the lower their acceptance of the CHC would be.

**Hypothesis 4 (H4):** Perceptions of challenges associated with entrepreneurship and innovation (EI) are positively related to the CHC. That is, the more the young people perceived challenges associated with EI in Hong Kong, the higher their acceptance of the CHC would be.

## Results

Given that mixed methods were adopted, the results are presented in two parts: (1) qualitative findings from the 83 focus group participants and (2) survey reports of the 1253 youth participants. Common themes from the qualitative method were extracted to develop items for the quantitative measure and to reveal the challenges and opportunities faced by young people in Hong Kong.

### Qualitative results

The results from the focus groups are presented according to the themes and subthemes (see Table 3), including challenges associated with entrepreneurship and innovation in Hong Kong, housing challenges (HC), socio-economic challenges (SEC), and Mainland China–Hong Kong Convergence (CHC).

#### Challenges associated with entrepreneurship and innovation in Hong Kong

Several participants mentioned that innovation and technology industries could be a way for them to obtain upward socio-economic mobility (*N* = 5). Also, some participants wanted to start a business or identify themselves as entrepreneurs (*N* = 11). Participant 1.2 observed that young people tended to start their careers as entrepreneurs during economic restructuring because they found it more difficult to move up the social ladder in this period. Furthermore, the rapid development of the Internet and technology has provided a favourable business platform for easier entrepreneurship. Hence, young people were more willing to take risks and start businesses to enhance their upward social mobility.

However, many participants thought that the Hong Kong government does not have substantial policy support and long-term planning for developing innovation and technology industries (*N* = 11), resulting in innovations being nipped in the bud. Meanwhile, participants added that Hong Kong does not have the right conditions for developing innovation and technology industries because not much attention is paid to scientific research, there is a lack of talent and a lack of land, and it does not meet the requirements for large capital investment and long-term returns:

Even if there were some investors, the fund would be very small or even unable to support the project. (9.7)

Furthermore, the participants believed that some of the laws and regulations in Hong Kong restrict creativity, innovation, and the development of technology, including “Uber” and financial information technology (*N* = 3). Restrictions in the legal system also increase the cost of operations, meaning that the development of new industries and the creativity of young people are stifled.

Even if a business can be started, the participants believed that maintaining the business is difficult (*N* = 8), especially when young people have limited capital and networks (*N* = 14):

Entrepreneurship is only a dream if you don’t have the networks and resources to promote your own ideas. (6.7)

New and small businesses find it very difficult to compete with large corporations or consortia that have established their capital and networks in the market (6.7). As a result, the scope for young people to start their businesses is significantly reduced, thus hindering their opportunities for upward socio-economic mobility.

Moreover, unaffordable high rent was cited as the biggest barrier to starting and maintaining a business. Participants (*N* = 6) mentioned that landlords aggressively and maliciously raise rents when they see successful entrepreneurs:

Some shops become famous when online pages and magazines interview them. Then, their landlords increase the rent beyond their profit, often resulting in the rapid closure of these profitable businesses. (2.10)

The landlords sometimes do not have a financial need but uphold a notion that ‘since you’re still doing well under the high rent, of course, I can increase the rent’. (8.6)

Rising rents have significantly reduced the profitability of entrepreneurs and have even led to the termination of businesses.

Regarding innovation in Hong Kong industries, the participants commented that Hong Kong has a single industrial structure and an overreliance on the financial, real estate, and service industries (*N* = 17). The overreliance on hoarding and speculative activities creates pseudo-economic growth and reduces the room for developing new technologies, inventions, and industries (9.9). Participant 8.6 further mentioned that “*there is no room for fantasies in the future while working in society, but merely survival*”, highlighting that young people can only take up relatively stable jobs expected by mainstream society instead of initiating start-ups. Hence, participants perceived a lack of innovation in the current Hong Kong economy.

Besides, participants felt that Hong Kong’s economy and assets had been monopolised by large consortia and the older generation (*N* = 8), who control the economic underpinnings and capital (*N* = 6):

Since all of them [the ruling class of Hong Kong] have to defend their interests, how can we believe that they will help develop new industries that affect their interests? (3.6)

Large consortia have taken over all the shopping malls and markets, and there are no small businesses anymore. (9.8)

Certain business tactics for controlling market shares had been observed by participants, such as consortia offering their services or products at extremely low prices through extensive distribution networks. Customers undoubtedly favour lower prices and patronise large enterprises, making it even more difficult for new entrepreneurs to survive. Participant 3.2 elaborated that the consortium owners assign their relatives or trusted aides to manage profitable projects. As a result, even if the younger generation initiates a business, they find it difficult to move up in the social hierarchy.

#### Housing challenges (HC)

The housing issue was stressed as the most important challenge (*N* = 9). Although some participants perceived housing as a basic human need (*N* = 11) or a tool for speculation (2.10), owning property was regarded as a kind of upward socio-economic mobility (*N* = 8):

If you don’t have a property, you have to rent an apartment. The rent will push you back into poverty. (5.6)

However, the participants also believed that the current policies could not protect the rights of Hong Kong citizens (*N* = 3). Property prices rose alongside the influx of hot money from the mainland (*N* = 4), so Participant 2.7 believed that the government should increase the stamp duty on property trades by non-Hong Kong residents. Full implementation of “Hong Kong property for Hong Kong people” was called on to protect the rights of Hong Kong citizens (*N* = 2).

Some (*N* = 8) mentioned increasing the proportion of subsidised housing, including public housing and housing under the Home Ownership Scheme, to over 60% of the total housing supply. In the current situation, young people with stable employment have been excluded from the waiting list for public housing. Individuals have to take on low-paid jobs to ensure their eligibility to apply for public housing (*N* = 3).

To get public housing, I would rather have a lower salary or adopt various means to lower my apparent salary in order to meet the criteria for application. (1.7)

Hence, the government was urged to match the prices against the median household income rather than the market prices. Participants believed that the people suffering most are those who exceed the income and asset limits for application but cannot afford private housing, most of whom are members of the younger generation (*N* = 6).

Another reason for the housing supply shortage is developers’ excessive hoarding. The government’s fault is reflected in the tilted policies favouring developers without any regulatory intervention (*N* = 2). Hence, the government should set more restrictions and conditions for developers regarding property supply and size (*N* = 3). To alleviate the housing challenges the younger generation faces, the government should increase the supply of subsidised housing as the long waiting time is unacceptable (*N* = 7); the government is urged to support homeownership for the young generation.

#### Socio-economic challenges (SEC)

The participants commented on the societal and economic challenges in Hong Kong. Some expressed a sense of helplessness and described the situation as playing in a “dead-end maze” (4.9) and being trapped in a loop of hope and disappointment (*N* = 2). Participant 4.5 explained that the sense of hopelessness results from the uneven distribution of resources. The unfairness was perceived in various settings, including land, housing, political participation, education, and cultural and recreational resources (*N* = 9).

##### Social challenges

Apart from personal difficulties, the participants faced a lot of social challenges. A few participants (*N* = 8) commented that Hong Kong has no room for them to develop their own thoughts. Participant 3.1 described himself as a screw in a large machine (society). Although he had thoughts, he could not do anything. Unrealistic social expectations make young people unable to express their true thoughts:

Hong Kong, as a capitalistic city, values money the most. Humanities or other innovative ideas have never been valued. Hong Kong is not a place where these non-profitable ideas are valued. (6.8)

Society keeps pushing students all the way down a path that we don’t really want. After graduation, I cannot find a life goal or am forced to do something I don’t want to because ‘everyone is doing it, so I have the pressure to do it’. (8.4)

Those humanitarian thoughts that are incompatible with capitalist values may be suppressed. The monopolisation and monotonous industrial structure force young people to work for the consortia in the financial industry:

Except for the financial and real estate sectors, it is very difficult to have good prospects in other industries. (7.7)

Without a diverse industrial structure, young people are forced to study related subjects to get into these industries and work for the consortia (*N* = 2). As a result, a vicious cycle has been created, and the industrial structure has become less varied (4.10). Under these circumstances, young people are resigned to putting aside their true desires to fulfil social expectations:

Society forces us to give up our interests and only requires us to do well in our study. We are all restricted by limited choices. (4.9)

There is always a notion in our mind that we should ‘forget about it and resign ourselves to it. Yes, we’re dissatisfied, but we’re going to do it anyway’. (6.4)

Hong Kong society has already established a life pattern or goal for young people, such as having a luxury car, a private flat, and an elite college qualification (7.6). Education seems to shape them further to follow these social expectations (*N* = 3).

Realistically, some participants believed that maintaining their livelihood outweighed putting their dreams and interests into practice (*N* = 5). Simply put, salary considerations are usually prioritised over a dream job (7.1). Because of low salaries, Hong Kong youths have little left over after deducting their daily needs, which further disables them from living in the ways they desire (*N* = 11). The huge life pressure generates struggles and difficulties:

It is always impossible for us to catch up with inflation, so we suffer so much from the pressure in life. (2.10)

On the one hand, I have worked hard to earn money but spent it all in the blink of an eye. On the other hand, it’s like upward mobility being impeded without savings. (2.2)

Participant 1.4 pointed out that low salaries are more prevalent among the younger generation. Low salaries delay or stop young people from proceeding to their next life stage, such as marriage or homeownership (*N* = 2). To some youths, the unfriendly business environment does not allow them to earn an income proportional to their efforts (*N* = 6):

Only if our boss appreciates our performance, we gain an opportunity. Appreciation is much more important than our ability. (7.1)

Low salaries do not enable young people to meet their living costs or enhance their living standards. The quality of life is not rising but declining. Moreover, educational background (even for PhD holders and elite college graduates) cannot guarantee a proportional income and secure job status but a heavy tuition liability (*N* = 2). Furthermore, the older generation and parents are also a source of suppression for some young people. Seniors do not value dreams, interests, entrepreneurship, and innovation as much as the younger generation (*N* = 8), thus causing tensions. Even if young people would like to realise their dreams, they would still feel responsible for their parents and significant others:

People will keep asking, ‘have you found a job yet?’, ‘how is your job search going?’, ‘what kind of work have you found?’ or ‘I don’t think it’s reasonable to earn $10,000 and something at the age of 30’. (6.8).

The concerns of money and family will always be there. (8.5)

Because of the discrepancy in thoughts between the two generations, young people cannot do what they truly desire.

Some participants perceived a difference between the current and past Hong Kong. They said that they could not enjoy the same freedoms as before, even though Hong Kong still enjoys the freedom of speech and assembly (*N* = 2). They were not satisfied with the performance of Legislative Council members and felt displeased and suppressed (*N* = 8), especially when they saw filibusters in the Legislative Council. They perceived the arguments and disturbances in the meetings as a farce, wasting time and money (*N* = 4). By contrast, more participants believed that the Legislative Council members merely make a noise to arouse public awareness and defend their voters’ voices (*N* = 14). The filibuster, commonly used in foreign countries, was regarded solely as a tactic in council meetings (*N* = 3), especially when the political party was powerless (*N* = 2). Therefore, the filibuster was seen not only as a source of suppression but also as a symbol reflecting the degree of suppression.

Hong Kong was known as the Pearl of the Orient, but the participants (*N* = 13) perceived that Hong Kong has gradually lost its specialness, including its local culture, creativity, human rights, freedom, and the rule of law (*N* = 3). The blind boast of foreign cultures, the homogeneous style due to the monopoly of consortia, and the loss of economic competence were perceived as the key factors leading to this fading away (*N* = 4). The CHC also brings risks to the unique cultural status of Hong Kong, further raising the importance of maintaining and promoting Hong Kong’s specialness (8.3).

##### Economic challenges

The government is accused of being a driving force behind an uneven distribution of social resources due to implementing economic policies that benefit the consortia, resulting in their expansion (*N* = 5). Although participants revealed tactics for contending with this challenge, such as frequently or only shopping at small businesses (*N* = 2), this was seen as only a drop in the ocean. The consortia still greatly affect the livelihoods of citizens. Consequently, small businesses cannot compete with the consortia (*N* = 6), resulting in high prices for daily necessities.

In addition, participant 7.6 perceived a decline in Hong Kong’s international economic status because of a lack of direction. The speculative economy and a lack of a real economy in contemporary Hong Kong further decrease its international competitiveness (8.3). Along with the implementation of the Individual Visit Scheme, services and travel were forced to cater to mainland visitors for profit (6.4), but the consequence was the eradication of other businesses with local features (*N* = 2). Ultimately, the scheme produced a fatal outcome for local businesses (3.6). Under its flashy surface, Hong Kong has gradually lost its importance in the global market (*N* = 2).

On the other hand, economic “Mainlandization” was also foreseen by the participants. The original localised features of Hong Kong have gradually been replaced by Mainland Chinese features. Participant 7.4 thought Hong Kong had been forced to follow the “China Model” or “The Beijing Consensus”, causing the loss of Hong Kong’s uniqueness. Indeed, the participants (*N* = 6) noted China’s distinctive economic development, which might even surpass Hong Kong’s:

Build Your Dreams, Tencent, and Huawei are slowly overtaking Hong Kong’s high-tech industries. I hope Hong Kong can find enhancement opportunities and a new role in the international market. (2.7)

If we don’t retain our characteristics or our way of doing things, we cannot have a place in the international market. (3.4)

Therefore, learning from Mainland China but maintaining the localised features of Hong Kong was believed to be a means to strengthen the business competitiveness of Hong Kong. However, no such policy was observed in the current adversarial situation. Because of the various social and economic challenges, the young generation has experienced suppression and felt despondency about upward mobility.

#### Mainland China–Hong Kong Convergence (CHC)

Participants admitted the relationship with Mainland China was an opportunity to promote their upward socio-economic mobility, but not everyone could grasp this opportunity. Their responses demonstrated three ways of accepting CHC, namely acceptance of the Chinese identity, cross-border living and working, and convergence.

##### Acceptance of Chinese identity

In terms of accepting Chinese identity, the national identity and the understanding of conditions in Mainland China were the major concerns. Several participants preferred a Hongkonger identity to a Chinese one. This sense of non-acceptance of Chinese identity resulted from an overemphasis on Hongkongers’ identity, a lack of recognition of Mainland China’s surpassing development, and an admiration of foreign products (*N* = 3). It is undeniable that Hong Kong citizens became Chinese after the handover of sovereignty from Britain to China (*N* = 8). Although people have to get used to a Chinese identity, CHC could be a good way for each to bring out the best in the other (1.4). A better understanding of the socio-economic conditions of Mainland China helps people evaluate convergence more objectively. The stigmatisation and intuitive resistance were regarded as meaningless, and people were reluctant to accept historical facts (*N* = 2). According to Participant 8.3, “as we are all Chinese, we have lots of shared and common views and beliefs”, and so the recognition of Chinese identity could be enhanced when there is more respect and autonomy, preferential policies, cultural interactions, business opportunities and benefits for local developments (*N* = 11).

Nevertheless, differences between Mainland China and Hong Kong still exist regarding language, the legal system, business models, lifestyles, politics, and cultures (*N* = 15). To mediate these differences, participants called for someone who could look from both sides to bring hope to handling the social cleavages in Hong Kong and enhancing the acceptance of Chinese identity (*N* = 2).

##### Acceptance of cross-border living and working

CHC has also brought opportunities for cross-border living and working. More and more people are choosing to live on the mainland (*N* = 4), and some participants stated that they would consider working on the mainland (*N* = 7). Given that cross-border working is a common phenomenon (7.5), participants pointed out that Mainland China has more social and financial resources and opportunities for Hong Kong youths to develop their careers and start-up enterprises, such as cheaper rent and more space and resources for entrepreneurship and innovation (*N* = 13). Although the rapid development of Mainland China threatens the economic status of Hong Kong, Hong Kong has still accumulated more soft skills and professional experiences throughout hundreds of years of social transformation and development (*N* = 3). These advantages can help Hong Kong youths to succeed on the mainland. Compared with mainland youths, the participants believed Hong Kong youths still have certain strengths that are attractive to mainland companies or can enhance their entrepreneurship:

You can find talent for certain industries or characteristics in Hong Kong, such as management personnel. Hong Kong has a particular work style, including punctuality, efficiency, politeness, and decent appearance. (7.2)

We have certain beliefs and values that help us start up our business, such as reliability, credibility, and being well-mannered. (7.4)

On the other hand, there are more job vacancies on the mainland that Hong Kong youths can explore if they can work humbly (5.8). Staff benefits in mainland companies could be even better than those in Hong Kong companies, especially when more international enterprises establish their offices in first-tier cities (*N* = 4). Working on the mainland can be a way for the young generation to move upward.

##### Acceptance of convergence

The CHC offers certain benefits to Hong Kong citizens, including online shopping, movies, food, and other entertainment (3.2). Some participants opined that many opportunities for economic development had been created during the convergence (*N* = 6). China has provided various preferential policies and infrastructure to benefit Hong Kong from money inflows, such as the high-speed rail Hong Kong Section and Stock Connects (2.5). Moreover, the rapid development of large online business enterprises in China has become a reference for Hong Kong youth to learn from and catch up with advanced business models (*N* = 4):

Hong Kong people used to be ambitious, dominant, and skilful, but now, they are merely entirely uncompetitive. Hong Kong people have less Fintech knowledge, bravery, aggressiveness, fluent language, and business capability. (5.4)

If Hong Kong youths can grasp opportunities from the convergence and fulfil their ambitions, they might find ways to move upward (*N* = 2). In particular, those mainland students studying in Hong Kong are the most talented and outstanding youths. They are in an advantageous position because of their fluent language skills, endurance, and social networks on the mainland (*N* = 3). Some participants (*N* = 5) believed that crises created opportunities by providing healthy competition to drive the Hong Kong youth to work harder and be aware of their own deficiencies. The competitions motivate Hong Kong youth towards self-enhancement. If we can merge the strengths of the youths in both places, the CHC would bring more opportunities than challenges (9.9).

In sum, the participants described the challenges associated with entrepreneurship and innovation, housing challenges, and social and economic suppression as the challenges to upward socio-economic mobility in Hong Kong, while the CHC is regarded as a new opportunity.

### Quantitative results

#### Measurement tools

Based on these qualitative data, a questionnaire was constructed to investigate and measure these four aspects on a five-point Likert scale (see Table 5), ranging from 1 (strongly disagree) to 5 (strongly agree), as well as to test the four hypotheses set out above.

**Table 5:** Item-level descriptive statistics for the four aspects (*N* = 1253).

Items	Mean	Standard deviation	Skewness	Kurtosis
*Challenges associated with Entrepreneurship and Innovation (EI)*				
1 I hope that the government will encourage the development of innovative technology industries through rent and tax concessions.	3.93	0.66	−0.51	1.13
2 I hope that the government will attract local and foreign companies to set up high-end industries in Hong Kong.	3.89	0.67	−0.36	0.62
3 I hope that the business community will create more entrepreneurial information and technical support platforms for young entrepreneurs.	4.01	0.65	−0.38	0.66
4 I hope that industry will become more diversified, jobs will increase, and young people will be able to find jobs.	4.19	0.65	−0.52	0.73
*Housing challenges (HC)*				
1 I hope that the government will do its best to prioritise the interests of Hong Kong people.	4.43	0.68	−1.10	1.39
2 I hope that the government will increase stamp duty for non-Hong Kong residents buying and selling land and property.	4.23	0.85	−0.87	0.12
3 I hope that the government will fully implement the “Hong Kong People Hong Kong Land” policy.	4.06	0.84	−0.61	−0.01
4 I hope that the government can link the price of Home Ownership Scheme flats with the median household income ($25,000 per month [2016 figures]).	3.81	0.81	−0.33	0.01
5 I hope that the government will increase the proportion of government-subsidised housing (public housing and Home Ownership Scheme housing) to 60% of new housing.	3.97	0.79	−0.52	0.35
6 I hope that the government will restrict the size of the units built by developers in the land lease when selling land.	3.80	0.83	−0.39	0.19
*Socio-economic Challenges (SEC)*				
1 My own thinking cannot fit with Hong Kong’s current values.	3.06	0.89	0.16	−0.34
2 The freedom of speech of Hong Kong people is not the same as in the past.	3.55	1.00	−0.44	−0.55
3 I hope that members of the Legislative Council will continue the filibuster.	2.85	1.10	0.12	−0.48
4 Hong Kong is not a suitable place for young people to pursue their dreams.	3.70	0.99	−0.45	−0.45
5 I think the older generation cannot understand my difficulties.	3.10	0.96	0.08	−0.70
6 My salary is not sufficient to meet my needs in life.	2.95	0.99	0.22	−0.75
7 Hong Kong is a place where income is not proportionate to efforts made.	3.53	1.01	−0.38	−0.51
8 Hong Kong has gradually lost its local characteristics.	3.81	0.92	−0.69	0.29
9 Hong Kong’s international characteristics are gradually being obscured by the Mainland.	3.73	0.91	−0.52	−0.17
10 Hong Kong’s economy has lost its competitive edge.	2.80	0.90	0.29	−0.56
11 Hong Kong lacks a real economy (such as industry, manufacturing, etc.), and is dominated by speculation.	3.73	0.84	−0.51	0.08
12 Hong Kong is a place where resources are unfairly distributed.	4.03	0.86	−0.74	0.44
13 Hong Kong’s economic decisions are mainly made in favour of large consortia.	4.13	0.74	−0.61	0.36
14 Small businesses in Hong Kong cannot coexist with large companies.	3.72	0.94	−0.61	0.09
15 The Individual Visit Scheme simplifies Hong Kong’s industries.	3.56	0.94	−0.32	−0.46
16 Hong Kong’s economy and industries are not diversified, so I don’t have enough work options.	3.17	1.03	0.06	−0.99
*Mainland China–Hong Kong convergence (CHC)*				
1 I am Chinese.	3.32	1.09	−0.55	−0.19
2 Hong Kong people should know more about national conditions.	3.27	0.98	−0.43	0.01
3 The Mainland’s economic development is conducive to Hong Kong’s young people starting businesses on the Mainland.	3.06	0.96	−0.39	−0.56
4 I hope that when the government promotes economic cooperation between Hong Kong and the Mainland, it will increase employment opportunities for Hong Kong’s young people on the Mainland.	3.43	0.91	−0.46	0.28
5 I will consider working on the Mainland.	2.39	1.18	0.17	−1.20
6 I will live in a Mainland city near Hong Kong.	1.99	1.06	0.73	−0.56
7 Hong Kong should accelerate its convergence with the Mainland. Otherwise, it will miss out on the development opportunities in the Mainland.	2.83	1.01	−0.12	−0.60
8 The competition from Mainland students can encourage Hong Kong youths to work harder.	2.67	0.98	0.09	−0.83

##### Challenges associated with Entrepreneurship and Innovation (EI)

Regarding EI challenges, the qualitative study revealed two major concerns: limited capital for young entrepreneurs and the lack of variety in industries. Hence, four questions were constructed that measured the challenges associated with entrepreneurship and innovation, which has four unidimensional items measuring the demand for improving the industrial structure. Items 1 and 2 assess the demand for fostering high-tech industries, whereas items 3 and 4 assess the demand for developing various entrepreneurial and career opportunities for young people. The higher the EI score, the more the individuals perceive that Hong Kong’s industries are facing challenges regarding transformation because of insufficient EI.

##### Housing challenges (HC)

The participants also urged the government to increase the supply of subsidised housing and link the Home Ownership Scheme prices to the median family income. Punitive charges for non-local speculators and clear restrictions for developers were also suggested to curb private housing prices. HC comprises six unidimensional items measuring the demand to improve the housing policy. Items 1–3 assess the demand to guarantee local people’s interest in the housing policy, while items 4–6 assess the demand for lowering the housing policy’s standards. The higher the HC score, the more the individuals perceive that the current housing policy should be more beneficial for the local people.

##### Socio-economic challenges (SEC)

The younger generation perceived challenges concerning many social and economic aspects: hindrance of their thoughts and dreams, incomprehension of the older generation, declining freedom of speech, incomes that are insufficient and disproportionate to their efforts, a fading of the local specialness, and filibustering as a symbol of suppression were all considered the sources of challenges. Moreover, the monotony of industries, loss of economic competence, the unfair distribution of resources, excessive speculation, and policies that favoured the consortia constituted economic suppression for the younger generation. SEC comprises 16 items that measure two dimensions: social and economic challenges. The higher the SEC scores, the more the individuals perceive themselves as being challenged by socio-economic issues.

##### Mainland China–Hong Kong Convergence (CHC)

Generally, the participants recognised that the mainlanders’ quality of life had improved and was even better than that of Hong Kong’s youth. They opined that there were greater business and career opportunities on the mainland, especially entrepreneurship opportunities. The CHC comprises eight items measuring three dimensions of perception relating to the convergence: acceptance of Chinese identity, cross-border living and working, and the CHC itself. The higher the CHC scores, the more strongly the individual perceives the advantages of the CHC.

#### Validity and composite scores of the four items

The above four items were generated from youth opinions expressed in the focus groups. They were developed not from existing theories, but from bottom-up induction to fit the local context. Item-level descriptive statistics for the four aspects were computed, and the normality of the data was assessed by skewness > ±2 or kurtosis > ±7 (Kim, 2013) (see Table 5). The internal consistency of each item, which is presented in Table 6, which ranged from 0.768 to 0.845. Hence, internal consistency among the items in the factorial structure was confirmed.

**Table 6:** Internal consistency and mean score of the four aspects (*N* = 1253).

Aspects	Internal consistency	Composite Score
Housing challenges (HC)	0.768	4.051
Challenges associated with entrepreneurship & innovation (EI)	0.845	4.003
Socio-economic challenges (SEC)	0.812	3.466
Mainland China–Hong Kong convergence (CHC)	0.818	2.829

Internal consistency is calculated by Cronbach’s *α.*

Table 6 shows the composite scores of the four items. The item “housing challenges (HC)” scored 4.051 out of 5, revealing that participants were concerned about insufficient affordable housing in Hong Kong and expected the government to develop a housing policy favouring the local people. Similarly, the item “challenges associated with entrepreneurship and innovation (EI)” scored 4.003, indicating that the participants believed that Hong Kong’s industry lacks diversity and EI, and thus needs restructuring. The “socio-economic challenges (SEC)” item scored 3.466, suggesting that participants tended to believe that they were being challenged by various socio-economic issues. Compared with the above three items of challenges, the participants scored lower (2.829) on the opportunity item “Mainland China-Hong Kong Convergence (CHC)”, indicating that they generally had a pessimistic view of the convergence. Yet, many participants agreed that economic cooperation between Hong Kong and Mainland China would increase employment opportunities on the mainland for Hong Kong’s young people (i.e., mean 3.43 out of 5; see Table 5, CHC 4).

#### Distribution of measures

**Fig. 2:**
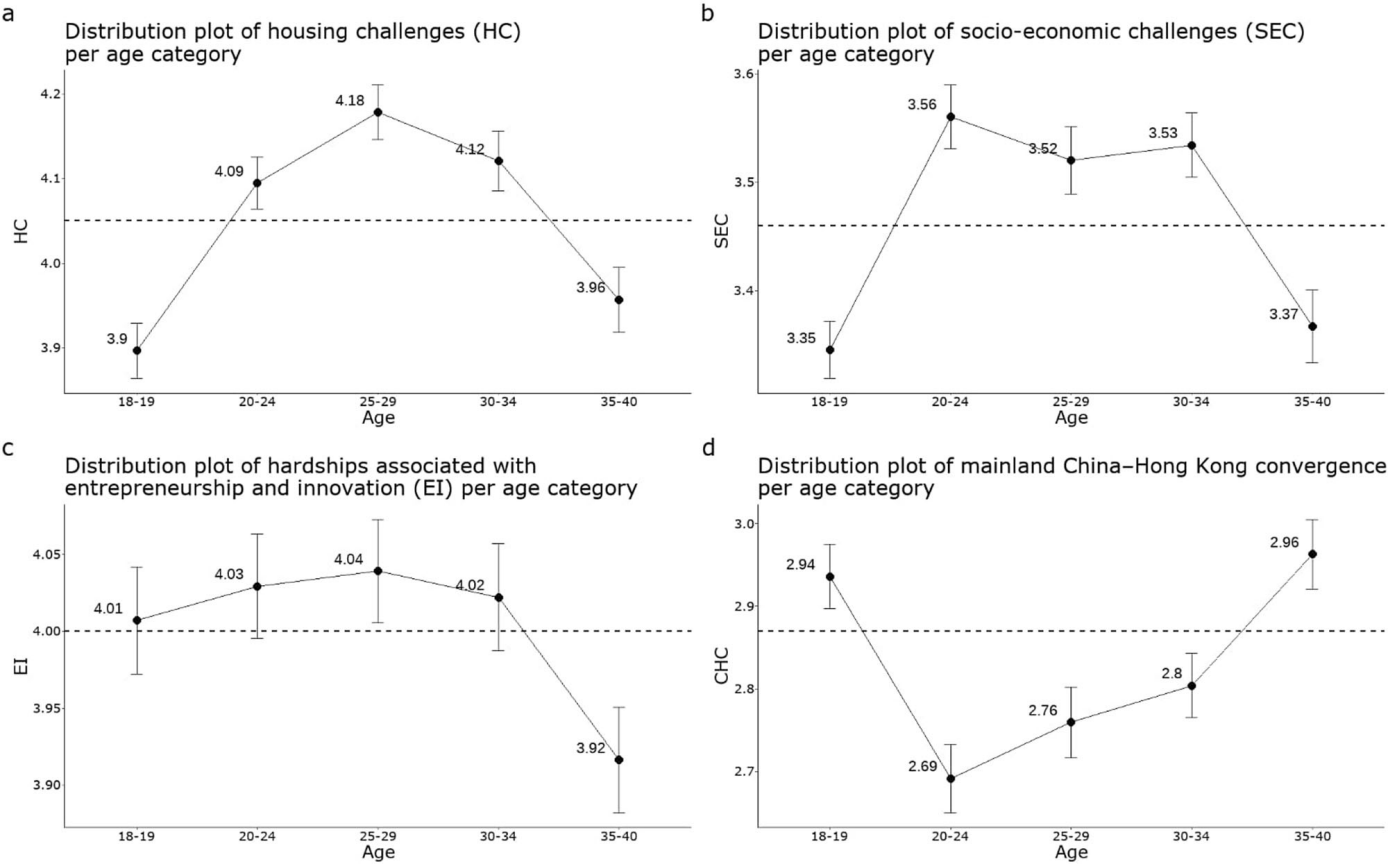
Distribution of housing challenges, socio-economic challenges, entrepreneurship and innovation, and Mainland China–Hong Kong convergence per age group. **a**–**d** The mean level of housing challenges (HC), socio-economic challenges (SEC), entrepreneurship and innovation (EI) and Mainland China-Hong Kong convergence by age group, along with their total mean level. Note: The black dashed line refers to the mean of a measure calculated with the overall sample. The dot refers to the mean of a measure calculated with a group within a specific age range. The error bar refers to the standard error of the mean.

The distribution of each measure by age group is presented in Fig. 2. The four measures largely follow a normal distribution pattern in different age groups. The mean values of the four measures by each age group do not apparently deviate from their corresponding overall mean. As can be seen from Fig. 2c, the means of the four age groups of EI largely overlap with the means of EI of the overall sample. Whereas the inter-group average value is the same when visually compared to the other three measures, the rightward distribution plot shows a jagged distribution pattern of EI, implying that the sample of each age group is more aggregated for certain scores on EI perceptions. In addition to the aggregation pattern in EI, the multiple outliers in EI, especially in the 36–40 age group that has upper-side outliers that are not shown in the rest of the age groups, indicate that participants vary in perceived entrepreneurial and career opportunities in terms of inter-group and intra-group. For HC (Fig. 2a), SEC (Fig. 2b), and CHC (Fig. 2d), the consistent distribution plots, stable mean and quartile positions, and sporadic occurrence of extreme values mark no notable differences among age groups for these three measures.

#### Ordinary least-squares regression

Ordinary least-squares regression (OLS)—a frequently used linear least-squares system to estimate undefined coefficients in a linear regression model—was applied to examine the relationships between the items identified. To examine the four hypotheses (H1–H4), an OLS model was implemented, while controlling for several socio-demographic variables. The intent here was to assess the explanatory power of the various challenges to the CHC.

**Fig. 3:**
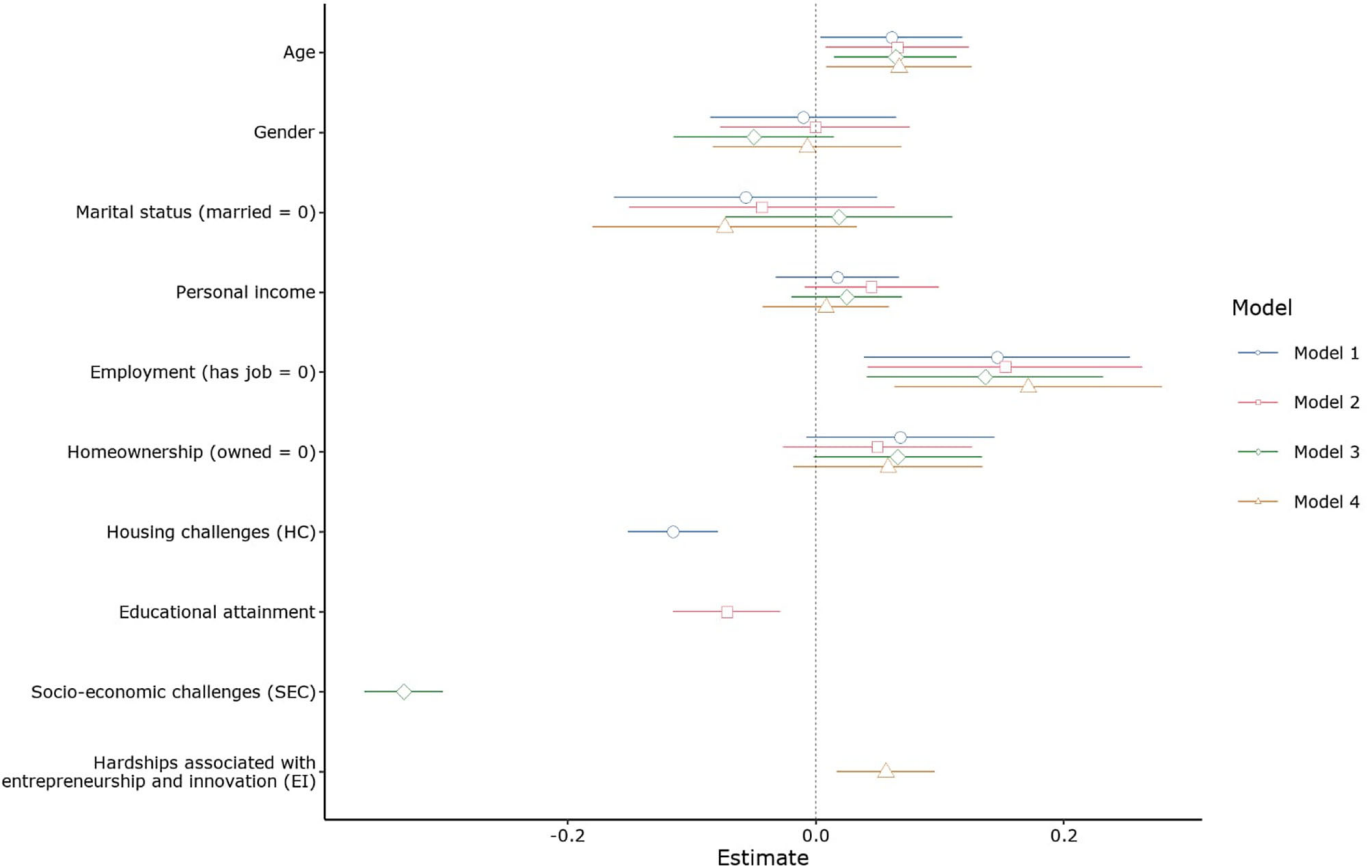
Effect plot for H1–H4 note. The line refers to a 95% confidence interval of the effect.

Table 7 presents the results of the examination of H1–H4, and Fig. 3 intuitively shows the effect sizes of all predictor variables on the dependent variable within the same plot scale. Two (age and employment) of the six control variables had a stable and significant effect on CHC though the effect from age can be dismissed because one side of the confidence interval of effect was much too close to 0. For H1, Model 1 shows the OLS regression of the HC effect on CHC. After controlling for six socio-demographic factors, a statistically significant negative effect of HC on the CHC was found (*B* = *−*0.21, *p* = <0.001), thus supporting H1. That is, young people would take a more negative view of the CHC if they faced housing challenges. The higher the pressure from housing, the lower their desire to engage in mainland life and work.

**Table 7:** Liner regression of housing challenges, educational attainment, socio-economic challenges, hardships associated with entrepreneurship and innovation on Mainland China-Hong Kong convergence.

	Model 1 (*N* = 1222)	Model 2 (*N* = 1221)	Model 3 (*N* = 1222)	Model 4 (*N* = 1222)
Age	0.01* [0.00, 0.02]	0.01* [0.00, 0.02]	0.01** [0.00, 0.02]	0.01* [0.00, 0.02]
Gender	−0.01 [−0.08, 0.06]	−0.00 [−0.07, 0.07]	−0.05 [−0.11, 0.01]	−0.01 [−0.08, 0.07]
Marital status (married = 0)	−0.06 [−0.16, 0.05]	−0.04 [−0.15, 0.06]	0.02 [−0.07, 0.11]	−0.07 [−0.18, 0.03]
Personal income	0.01 [−0.01, 0.03]	0.02 [−0.00, 0.04]	0.01 [−0.01, 0.03]	0.00 [−0.02, 0.03]
Employment (has job = 0)	0.15** [0.04, 0.25]	0.15** [0.05, 0.26]	0.14** [0.05, 0.23]	0.17** [0.07, 0.28]
Homeownership (owned = 0)	0.07 [−0.01, 0.15]	0.05 [−0.03, 0.13]	0.07 [−0.00, 0.13]	0.06 [−0.02, 0.14]
Housing challenges (HC)	−0.21***[−0.28, −0.15]			
Educational attainment		−0.05***[−0.09, −0.02]		
Socio-economic challenges (SEC)			−0.69***[−0.75, −0.62]	
Hardships associated with entrepreneurship and innovation (EI)				0.11** [0.04, 0.17]
*F* statistic	*F*(7, 1214) = 8.89	*F*(7, 1213) = 4.86	*F*(7, 1214) = 65.04	*F*(7, 1214) = 4.6
*R*^2^	0.05	0.03	0.27	0.03
Adjusted *R*^2^	0.04	0.02	0.27	0.02

The statistical value of four linear regressions is formatted with a pattern of regression coefficient *p*-value [lower bound of confidence interval, higher bound of confidence interval].

***p < 0.001; **p < 0.01; *p < 0.05.

The bold values refer to the model support for the four corresponding hypotheses.

The OLS regression results for the effect of educational attainment on CHC (examination of H2) are shown in Model 2 in Table 7. The results are largely comparable to the results shown in the examination of H1 but with a smaller effect size. Educational attainment had a significantly negative effect on it, suggesting a higher education level predicts lower acceptance of the CHC (*B* = −0.05, *p* < 0.001). As such, H2 is supported. Although there was a significant effect, the bound of confidence interval close to 0 ([−0.09, −0.02] and Fig. 3) reveals that the effect is weak and might lose its significance once a strong predictor is added to the model.

The examination of H3 is reported in Model 3 in Table 7. The CHC was regressed on SEC after controlling for age, gender, marital status, personal income, homeownership, and employment. The effect of SEC on the CHC reached a significant level (*B* = −0.69*, p* < 0.001). Results show that the young people’s perceptions of SEC were negatively related to the CHC. That is, the more the young people perceived challenges in their socio-economic conditions, the lower their acceptance of the CHC. Thus, H3 is supported. The effect is much stronger than other predictors (Fig. 3).

In contrast to the previous three hypotheses, the examination of H4 in Model 4 in Table 7 reveals that young people’s perceptions of EI challenges were positively related to the CHC (*B* = 0.11*, p* < 0.01), suggesting that the more the young people perceived challenges associated with entrepreneurship and innovation, the higher their acceptance of the CHC. Hence, H4 is supported.

## Conclusion

The present study has revealed that young people face a number of challenges in their daily lives during the CHC. Although these youth’s comments and views were collected in 2017, the findings are still solid to contribute to the understanding of the challenges and opportunities presented to Hong Kong’s youth. According to Du and his colleagues (2021), socio-economic mobility is shaped by past experiences of mobility. With a better understanding of the youth’s perception of socio-economic mobility, a more concrete and fair explanation of future mobility can be projected (Du et al., 2021).

In short, opportunities for entrepreneurship and innovation are limited because of the existence of monotonous industries. Moreover, young people work in jobs with incomes disproportionate to their efforts; their living spaces are limited, resulting in high rents and housing prices, not to mention the lower possibility of homeownership. They, therefore, want the government to increase the supply of subsidised and affordable housing and to regulate speculators and property developers. Furthermore, many young people regard Hong Kong as a place that discourages them from having their own dreams. Unfair distribution of social resources and consortia-favoured business policies have constituted the economic suppression of the young generation. While they face strong competition from mainland youth, they recognise that their counterparts’ quality and capabilities have been improving and are even better than theirs. Simultaneously, they agree that there are greater business, career, and entrepreneurship opportunities on the mainland, even though they are pessimistic about the convergence.

To conclude, the concerns of Hong Kong’s young people comprise (1) the economic structure, which largely favours the development of large enterprises over local small businesses, and the lack of diversity in industries, which leads to challenges in providing more entrepreneurship and innovation opportunities; (2) high rent, which constitutes a barrier for running a business; (3) high property prices, which affect young people’s ability to afford a flat or build their own home; (4) receiving a salary that only accommodates their basic needs in life; (5) societal culture, with little freedom or autonomy to express their own voice fully and pursue their preferred pathway; and (6) attaining a high level of education, which does not guarantee socio-economic progression (Lee, 2016) and is affected by the economic and socio-cultural conditions of the society.

Conversely, some young people acknowledged the advantages of the CHC, wherein people could enjoy more social resources, cheaper rents, lower wages, and more scope for entrepreneurship and innovation, which constitute opportunities for career development among Hong Kong’s youth. Nevertheless, their tendency to live and work on the mainland is not high, mainly because those who have a high level of education and experience housing and socio-economic challenges may feel distressed about the convergence, which hinders them from embracing such opportunities.

This suggests that it is important to strike a balance between the features and cultures of the two places so that the CHC can maximise the advantages and opportunities of both places rather than creating challenges that threaten Hong Kong’s competence and uniqueness. For instance, the Mainland and Hong Kong Closer Economic Partnership Arrangement boosted Hong Kong’s economy after the SARS pandemic in 2003 (Nagy, 2015). Therefore, enhancing the CHC can be a future priority for policymakers while it remains important to consider young people’s life experiences and internal sense—whether they perceive every person as having reasonable opportunities for socio-economic progression using their own abilities and efforts (Alesina et al., 2004)—so as to handle social dissatisfaction. More policies to deal with housing demands, job opportunities, and entrepreneurship should be introduced to reduce the conflict with mainlanders, thus enhancing the acceptance of convergence.

The CHC, operating under the principle of “One Country Two Systems,” is unique in the world. Hong Kong is a self-administrative region of China, and people from both places have maintained regular social and business interactions for decades; hence, the people of Hong Kong are not refugees. Thus, the guiding principles of the United Nations Refugee Agency on refugee integration into a host country cannot be applied here (United Nations High Commissioner for Refugees, n.d.). Yet, the experiences of the people of Hong Kong mark a critical social transformation between the two social systems. How the government handles the situation, what influences young people’s perception of society and governance, and how to maintain social stability throughout the transformation are of vital importance to countries worldwide to understand the measures for smooth and stable social transformation. Policies relating to “reducing privileges of interest groups and closing the gap between individual efforts and success” (Bjørnskov et al., 2013, p.91) help maintain a social structure that young people perceive as providing adequate opportunities for their progression. If the policies are maintained in a well-balanced, mutually beneficial way during the convergence, young people will be more willing to embrace opportunities and face challenges brought about by the convergence, thereby resulting in a more harmonious society and socio-economic progression.

## Supplementary information

Dataset 1

## Data Availability

The datasets analysed during the current study are available in the Dataverse repository: 10.7910/DVN/MTNOQN.
